# A mixed methods study of how clinician ‘super users’ influence others during the implementation of electronic health records

**DOI:** 10.1186/s12911-015-0154-6

**Published:** 2015-04-10

**Authors:** Christina T Yuan, Elizabeth H Bradley, Ingrid M Nembhard

**Affiliations:** Department of Health Policy and Management, Yale School of Public Health, New Haven, USA

**Keywords:** Electronic health record (EHR), Super users, Implementation, Social influence

## Abstract

**Background:**

Despite the potential for electronic health records (EHRs) to improve patient safety and quality of care, the intended benefits of EHRs are not always realized because of implementation-related challenges. Enlisting clinician super users to provide frontline support to employees has been recommended to foster EHR implementation success. In some instances, their enlistment has been associated with implementation success; in other cases, it has not. Little is known about why some super users are more effective than others. The purpose of this study was to identify super users’ mechanisms of influence and examine their effects on EHR implementation outcomes.

**Methods:**

We conducted a longitudinal (October 2012 – June 2013), comparative case study of super users’ behaviors on two medical units of a large, academic hospital implementing a new EHR system. We assessed super users’ behaviors by observing 29 clinicians and conducting 24 in-depth interviews. The implementation outcome, clinicians’ information systems (IS) proficiency, was assessed using longitudinal survey data collected from 43 clinicians before and after the EHR start-date. We used multivariable linear regression to estimate the relationship between clinicians’ IS proficiency and the clinical unit in which they worked.

**Results:**

Super users on both units employed behaviors that supported and hindered implementation. Four super user behaviors differed between the two units: proactivity, depth of explanation, framing, and information-sharing. The unit in which super users were more proactive, provided more comprehensive explanations for their actions, used positive framing, and shared information more freely experienced significantly greater improvement in clinicians’ IS proficiency (p =0.03). Use of the four behaviors varied as a function of super users’ role engagement, which was influenced by how the two units’ managers selected super users and shaped the implementation climate.

**Conclusions:**

Super users’ behaviors in implementing EHRs vary substantively and can have important influence on implementation success.

## Background

The electronic health record (EHR), defined as an electronic version of a patient’s medical history that is maintained by the provider over time [1], is increasingly recognized as an important tool for improving patient safety and quality of care [2-4]. Despite the potential of EHR use to improve the health of individuals and the performance of providers, significant barriers to its use remain [5-7]. One important subset of challenges relates to the implementation process [8,9], which involves gaining targeted organizational members’ skillful, consistent, and committed use of a practice [10].

To foster the successful implementation of EHRs, a common recommendation is to enlist super users [11], or individuals, typically already working within the organization, who receive extra training on the selected EHR system so that they can provide frontline technical support to their peer users [12]. Emerging research suggests, however, that leveraging super users during EHR implementation does not always contribute to implementation success [13]. Past research on super users, which is limited, has identified several key roles through which super users might enable implementation, including serving as project liaison between the EHR developer and clinical team [14], facilitator during EHR training sessions [15], role model for their colleagues [16], and technology “ambassador” who helps foster acceptance of change [14,17]. What remains unknown are the mechanisms by which super users influence implementation success at the point of user need and the impact of contextual factors, both of which might explain why super users are effective in some cases but not in others.

One of the primary reasons why super users are expected to have an effect on the implementation process is because of their potential for social influence. Social influence is the alteration of an attitude or behavior by one individual in response to another [18]. Social influence theories, such as social information processing theory [19] and social learning theory [20], posit that individuals’ beliefs and use of technology are influenced by the opinions, information, and behaviors of salient others with whom they interact [21]. Applying these theories to super users in EHR implementation, these theories suggest that the behaviors of super users – who are salient because of their formal role in a large change effort – influence the beliefs and EHR use of their peers, particularly in the early stages of the implementation process when employees are first learning how to use the EHR system in their day-to-day work. A growing body of evidence from the information systems literature indicates that learning new technology is deeply embedded in doing [22] and that learning is most effective when it occurs in the actual work context, in the presence of coworkers, and while performing routine job tasks [23]. Thus, the behaviors that super users use to support their peers, particularly during the ‘go-live’ support stage of the implementation process in which individuals are learning to use EHRs in real-time, should have a meaningful impact on implementation outcomes. It is in this critical juncture after the EHR ‘go-live,’ when strategies to promote user engagement can be the most powerful [24] that we ask: what are the specific behaviors that super users use to influence others, and ultimately, implementation outcomes?

In this paper, we present the results of a comparative case study in which we sought to identify the mechanisms of super users’ influence on implementation success and examine the impact of a core contextual factor: how super users are selected. Specifically, we compared super users’ behaviors and implementation outcomes in two units of a large, academic hospital implementing an EHR system from October 2012 – June 2103 using data gathered from surveys, in-depth interviews, and observations. The two units were chosen for comparison because they differed in how nurse managers selected super users (volunteered or appointed), which allowed us to assess behaviors in two contexts. Our data suggest that the reason why some super users foster implementation success more effectively than others lies in the behaviors that they use to support implementation, which varied as a function of their role engagement. Our findings provide insight that may help health care organizations better select, prepare and support super users so that they can realize their potential for positive influence on the implementation of EHRs, and health information technology broadly.

## Methods

### Study design and sample

We used a longitudinal (nine months), convergent mixed methods study design that simultaneously employed qualitative and quantitative methods [25,26] to conduct a comparative case study of super users on two medical units of a large, urban academic hospital implementing a new EHR system. Qualitative methods are well-suited for investigating complex phenomena, such as social influence, which are difficult to measure quantitatively [27] as well as topics with limited theoretical and empirical development, which require rich, detailed, and evocative data to generate new constructs or connections between phenomena [28]. We used two forms of qualitative research – observations and in-depth interviews – to identify the specific behaviors that super users used to influence others in the two units, and similarities and differences between units. Quantitative methods were then used to explore whether our qualitative findings were reflected in units’ implementation performance, using survey data collected before and after the EHR ‘go-live.’ Taken together, our qualitative and quantitative analyses served as complementary approaches [29] for developing a more complete picture of how super users influence others, and ultimately, the successful implementation of EHRs.

The two units in our comparative case study were similar with respect to important characteristics including size, employee demographics (e.g., age, gender, and years spent in the profession), and organizational context (e.g., intergroup relations between professional groups) (Table 1). The two units differed in terms of how super users were chosen; the process by which super users were selected is an important contextual factor that we sought to understand because it is likely to shape the ways in which super users may be perceived and hence interact with others on the unit. On Unit 1, a geriatrics unit, nurse managers asked super users to volunteer for the position. On Unit 2, a cardiology unit, nurse managers selected super users who they considered to be technologically savvy. Our study population included nurse super users, nurse managers, nurses, patient care associates, and secretaries who worked full-time on the two medical units. Given the longitudinal nature of the study and physicians’ frequent rotation schedule, physicians were not included in our sample. All research procedures were approved by Yale University’s Human Subjects Committee [Protocol #: 1208010692].

**Table 1:** **Baseline characteristics of units 1 and 2**

	**Unit 1**	**Unit 2**	**T value**	**P value**
	**mean (s.d.)**	**mean (s.d.)**		
***Demographics***				
Number of employees	42	48		
Number of super users	6	9		
% Female	93.55%	89.66%		
Age	32.69 (11.07)	38.21 (13.98)	1.67	0.10
Years in the profession	6.48 (8.24)	11.01 (12.25)	1.69	0.10
Years in the hospital	5.53 (8.35)	9.59 (11.55)	1.57	0.12
Hours worked per week	33.42 (10.14)	35.52 (8.38)	0.87	0.39
***Organizational context***				
Openness to change	3.63 (0.63)	3.84 (0.50)	1.42	0.16
Intergroup relations between professional groups	3.83 (0.59)	3.96 (0.52)	0.90	0.37

### Qualitative arm

#### Data collection

During the first six weeks of EHR implementation (January – March 2013), the first author observed all 15 super users across the two units (Unit 1: 6 super users; Unit 2: 9 super users). She also observed a purposeful sample of 9 non-super user clinicians (Unit 1: 3 clinicians; Unit 2: 6 clinicians) who were selected because of their perceived influence on the unit (as assessed by social network data collected in a pre-implementation survey, which is described later in the paper). Observations were conducted across all days of the week and all times of the day for a total of 115 hours. Each participant was observed on at least two occasions, with each observation period lasting approximately two hours. During the observation period, the first author collected extensive field notes about the interactions between super users and clinicians and the behaviors that super users used to support or hinder the implementation process. After each observation period, the first author reviewed the field notes and added reflective notes about emerging themes.

We then used in-depth interviews to reconcile our observations with participants’ own perceptions of how super users influenced implementation. Between March and May 2013, the first author interviewed a purposeful sample of 8 super users (Unit 1: 5 super users; Unit 2: 3 super users) and 16 non-super user clinicians (Unit 1: 7 clinicians; Unit 2: 9 clinicians) who were selected to ensure diversity with regards to age, shift (e.g., day, night), role (i.e., manager, super user, nurse, patient care associate, and secretary), and whether the participant had been previously observed (half were both observed and interviewed). We conducted 24 interviews because theoretical saturation was reached at that point (i.e., no new concepts emerged) [30]. The interviews averaged between 30–45 minutes and were conducted in the nurses’ break-room. We used a semi-structured discussion guide that began with a ‘grand tour’ question [31] to elicit a broad picture of the participant’s experience with the EHR implementation (i.e., “I am interested in hearing about your experience with the EHR implementation so far. What has gone well? What has not gone well?). We then asked participants to reflect on how super users influenced others, probing on super users’ behaviors (e.g., “Does [super user’s name] use any specific strategies or actions to influence others?”), characteristics (e.g., “What about [super user’s name] makes him or her influential?”], and the perceived impact of super users’ actions [e.g., “In what ways do you think [super user’s name] influenced you?”]. With interviewees’ informed verbal consent, interviews were tape-recorded and professionally transcribed.

#### Data analysis

We used the grounded theory approach [32] in which interview and observational data were reviewed line by line in detail. As a concept became apparent, a code or tag, was created and assigned to the relevant line(s). To ascertain whether a code was appropriately assigned, we employed the “constant comparative” method to compare text segments to segments that had been previously assigned the same code to see whether they reflected the same concept [33]. Through this process, the code structure evolved inductively, reflecting “the ground,” or experiences of participants. The codes and code structure were considered finalized at the point at which no new concepts emerged (i.e., theoretical saturation), at which point, the data were then re-analyzed to apply the codes from the finalized code structure. Several strategies recommended by experts in qualitative research were used to ensure the analysis was systematic and verifiable [34,35], including the creation of an audit trail of code development, triangulation of data from different sources, and the independent coding of data by two researchers who resolved differences through discussion. ATLAS.ti software was used to organize the data and facilitate its analysis. In reporting our qualitative findings, we have complied with the Standards for Reporting Qualitative Research (SRQR) [36].

### Quantitative arm

#### Data collection

We administered a pre-implementation survey 2–4 months (October–December 2012) prior to the “go-live,” or start date, of the EHR system and then administered a follow-up survey 3–5 months (April–June 2013) after the EHR go-live. The survey response rate was 67% (N = 60/90) for the pre-implementation survey and 78% (N = 63/81) for the follow-up survey; we had longitudinal data for 43 individuals (out of a maximum of 52 individuals who completed the pre-implementation survey and were still working on the units at follow-up). The survey assessed individuals’ demographics, social network ties (e.g., who they turned to for advice), and implementation outcome, which was measured by respondents’ self-assessed information systems (IS) proficiency. We ascertained IS proficiency, also termed perceived ease of use in the technology literature, using an established measure of the degree to which a person believes that using a system would be free of effort [37,38]. We chose to focus on IS proficiency because users with greater IS proficiency tend to use those systems more effectively and efficiently than users who are less proficient [39,40]. To measure IS proficiency, we used three items adapted from Kane and Borgatti [37]’s scale: “I find [the EHR] to be easy to use,” “Interacting with [the EHR] requires a lot of my mental effort” (reverse-scored), and “I find it easy to get [the EHR] to do what I want it to do.” Respondents reported their agreement with each item using a 5-point Likert scale (1 = Strongly Disagree; 5 = Strongly Agree). Cronbach’s alpha for the scale was 0.75, indicating that the scale had acceptable reliability [41].

#### Data analysis

We used multivariable linear regression to estimate the relationship between clinicians’ IS proficiency at follow-up (the dependent variable) and the clinical unit in which they worked (the independent variable). We adjusted for clinicians’ pre-implementation IS proficiency (covariate). Given the limited statistical power due to our small sample size (N = 43 clinicians), we did not include as covariates clinicians’ demographics (e.g., age, gender, number of years working in the profession), which did not vary by unit and thus were unlikely to confound the effects of interest.

## Results

### EHR implementation context in which super users operated

Super users operated within the context of a hospital-wide EHR implementation that had negative and positive aspects. In terms of negative aspects, participants on both units consistently mentioned challenges related to the technology itself, including problems related to the functionality of the EHR (e.g., not being able to log into the system) and the complexity of the EHR platform. A nurse manager from Unit 2 (ID11) highlighted this complexity when she said, “if you want to find out the orders for a patient, there are 10,000 different ways of doing it.” Many participants also voiced their dissatisfaction with the EHR training, which covered general principles of using the system but not the specifics required for their daily workflow. A nurse on Unit 1 (ID18) remarked: “The training itself was worthless. I learned pretty much everything that I learned by doing it.” Several participants attributed this lack of tailored training to the non-clinical backgrounds of most of the trainers and support staff that were contracted by the EHR company. For example, a super user on Unit 1 (ID10) reflected on the inability of the contracted staff to apply the technology within the clinical work environment:

“They really weren’t that helpful because they were mostly IT people so it’s not like they were clinical…They know how to navigate through [the EHR] and everything but as a nurse on the floor…they didn’t have any answers to their questions.”

Furthermore, several participants reflected on adverse changes in the interactions and communication patterns that arose from the EHR implementation. For example, a nurse on Unit 2 (ID5) expressed how the EHR created new relational dynamics between clinicians that placed a greater emphasis on work roles than interpersonal relationships:

“I always knew my physicians, they always knew me…now you’re looking for that familiar face or person; you’re going up to them and there’s no relationship. I think that’s sad about all the technology. I mean it’s faster and that’s great and I can get what I need…but then you never learn. Like “I can count on her, oh she’s on tonight, great.” Before you would build a relationship where you would trust each other. Now you’re like, “Whose who, what’s what? Who am I talking too? Who am I calling?” I guess that’s just the wave of the future. It doesn’t really matter; everybody’s equal is what they say. A nurse is a nurse. A doctor is a doctor. Just get what you need. It’s a little colder.”

Along with these challenges, participants identified several factors that positively affected implementation, including structured opportunities to share best practices on how to effectively use the EHR (e.g., webinars, tip sheets, cross-departmental meetings) and a highly engaged senior management that articulated a clear vision and was a visible presence on hospital floors during the EHR “go-live.” Having super users was perceived to be a particularly helpful strategy because it provided clinicians with someone to turn to for support, who knew their work, and with whom they were familiar, as evidenced by these illustrative quotations.

“I think that was just comforting for [staff] to know that if they had any problem someone was always there to help” (ID24).

“The super-user was a great idea…because they’re familiar with your own specialty. They’re able to key in and drill down the specific documentation that’s needed for our specialty… where the [the contracted support staff] were very generic” (ID2).

“Having the super users I think helped de-stress a lot of the people on our floor… because it’s one of us that they can come to and not just a stranger” (ID10).

### Super user behaviors that support implementation – shared across units

Participants highlighted key behaviors that super users on both units used to support implementation (Table 2). These supportive behaviors included: (1) reporting problems with the EHR to someone in a position to fix it [e.g., “So whenever somebody had a question and I couldn’t really answer it… I would present that problem to the [cross-departmental EHR] meeting to see if they could give me a faster answer than the ticket” (ID9)]; (2) employing teaching strategies that promoted “learning by doing” (e.g., letting the user control the computer mouse); and (3) providing extra support to individuals struggling with the change [e.g., “[the super user] did a one-on-one with [nurse’s name] every day for 2 weeks because she was so petrified” (ID5)]. Notably, these shared behaviors were emphasized in the hospital-wide training program for super users.

**Table 2:** **Super user behaviors shared across units**

**Super user behaviors that supported implementation**	**Super user behaviors that challenged implementation**
(1) Reporting problems with the EHR to someone in a position to fix it	(1) Losing patience with coworkers
(2) Employing teaching strategies that promoted “learning by doing”	(2) Losing track of what material was taught to whom
(3) Providing extra support to individuals struggling with the change	(3) Spreading negative opinions about the EHR
	(4) Creating workarounds that undermined the appropriate use of the EHR

### Super user behaviors that undermined implementation – shared across units

Super users on both units also engaged in behaviors that undermined implementation (Table 2). Particularly in the early stage of implementation, serving as a super user was physically and emotionally draining. Epitomizing this, a super user from Unit 2 (ID4) lamented, “If I hear my name called one more time, I’m going to have a breakdown.” At times, the weight of these demands manifested in the following unsupportive behaviors: (1) losing patience with coworkers [e.g., “I don’t think that she even realized that she started to get aggravated… you felt bad. You’d be like, ‘Oh, now I don’t want to ask her ‘cause she’s gonna get aggravated so you would stay away’” (ID5)]; (2) losing track of what material was taught to whom [e.g., “She was exhausted… she thought so many times she said this but still people didn’t know. She thought everybody knew it because she’d said it probably 15 times. But then there’s 50 staff” (ID12)]; (3) spreading negative opinions about the system [e.g., “[Super user’s name] does vent a fair amount and if there were frustrations that she had with [the EHR] you would get just as much direction as you would sort of her opinion and frustration about it as well” (ID23)]; and (4) creating workarounds that undermined the appropriate use of the EHR [e.g., “The only problem is a lot of the super users are young and so they’re very computer savvy, so they figured out workarounds. And then we weren’t doing [it] the right way” (ID5)].

### Super user behaviors that differed between units

Our analysis revealed differences in behaviors between units: super users on Unit 1, who volunteered for the position, used more effort-intensive behaviors to foster implementation success than super users on Unit 2, who were appointed by nursing management. Specifically, four key behaviors differed between units: (1) proactivity, (2) depth of explanation, (3) framing, and (4) information-sharing (Table 3).

**Table 3:** **Super user behaviors that differed between units**

	**Super user behaviors distinct to Unit 1**	**Super user behaviors distinct to Unit 2**
(1) Proactivity	Proactively supporting their peers	Reactively supporting their peers
(2) Depth of explanation	Emphasizing why actions had to be performed in the EHR	Demonstrating how to accomplish tasks in the EHR but not explaining the logic behind these actions
(3) Framing	Using positive frames to diffuse tension	Using neutral frames to diffuse tension
(4) Information-sharing	Consistently sharing information about the EHR with all of the clinicians on the unit	Limiting the spread of information about the EHR to individuals they interacted with the most

#### Proactivity

Staff on Unit 1 described how super users proactively supported the learning process by devising ways to expand on the EHR classroom training (e.g., developing checklists of common EHR tasks), offering assistance proactively [e.g., “Every 2 seconds she’d ask me do you need help with this or do you need help with that?…Every single shift I worked…she would go around asking” (ID24)], and encouraging others to actively learn [e.g., “I was sitting with the staff members and saying, show me how to do this, are you comfortable with this, do you have questions?” (ID9)].

Conversely, the super users on Unit 2 assumed a more reactive approach to supporting their peers. For example, when a super on Unit 2 was asked whether she proactively shared information about the EHR with others, she responded “Well people will come and find you.” In addition, respondents noted that some of the super users on Unit 2 were not cognizant of their needs [e.g., “Those two super users were just hanging around…they weren’t very attentive. I don’t know what they were doing.” (ID12)].

#### Depth of explanation

Even though there was evidence of learning by doing on both units, the super users on Units 1 and 2 differed in the extent to which they explained the rationale for various actions in the EHR. On Unit 1, the super users placed greater emphasis on making sure that their peers understood not just what was expected but *why* they had to do something in the EHR. For example, a secretary on Unit 1 (ID14) remarked: “[Super user’s name] just didn’t show me how to do it. She explained why it happened.” In contrast, on Unit 2, the super users showed their colleagues how to accomplish tasks in the EHR but did not explain the logic behind these actions. For example, a nurse on Unit 1 (ID5) shared, “my super user … he’d say you don’t need to know that.”

#### Framing

As noted previously, during the implementation process, staff on both units would regularly voice their dissatisfaction with the EHR. In an effort to diffuse tension, super users provided frames, i.e., “definitions of organizational reality that serve as vehicles for understanding and action” [42]. The frames differed between units. On Unit 1, super users largely used positive frames such as “[the EHR’s] great…I think it’s safer for the patients” (ID10). Conversely, on Unit 2, super users largely maintained neutral frames such as “you’ll get used to it” (ID16) or “it will be okay” (ID8).

#### Information-sharing

On Unit 1, super users consistently shared information about the EHR with all of the clinicians on the unit. A super user (ID4) explained their system of disseminating information, saying:

“If I learned something, I would share it. And if [the other super users] learned something, they would share it. And I would take it back to, because we have groups, like I have 4 or 5 people that I work with… So if [super users’ names] said ‘oh this is how we’re doing it in the computer system,’ I would go back and tell my 5 people.”

In contrast, on Unit 2, super users tended to limit the spread of information to individuals they interacted with the most, many of whom had pre-existing social relationships with the super user. As the assistant manager observed:

“I’ve seen… [the super users] kind of like you know, ‘oh this is my friend. I’ll spend more time with her than the rest of the other people,’ which is not right” (ID11).

By concentrating support on certain pockets of individuals, super users inconsistently shared information by spending more time with some individuals and neglecting others. In an effort to make the spread of information more systematic, the manager reflected:

“I’m thinking of having each super-user have a packet of the tips and then having a sign-off for their team so that we actually know that it’s being done because right now we share it but not everyone hears it” (ID2).

### Super user role engagement

Our analysis suggested that differences in super users’ behaviors between units stemmed from differences in role engagement, defined as “a positive, fulfilling, work-related state of mind characterized by vigor, dedication, and absorption” [43]. We found that super users’ engagement was shaped by whether they appraised the demands as *opportunities* or as *burdens*. On Unit 1, super users generally viewed the demands of the role as an opportunity to advance professionally (e.g., advancement in the clinical ladder program) or maintain social cohesion [e.g., “I feel like we all said this isn’t just me… this is all of us together helping” (ID4)]; and thus committed to using more intensive behaviors to support implementation. Conversely, on Unit 2, super users generally viewed the role as burdensome [e.g., “I’ve never talked so much in my life…for me it was very aggravating because all the questions I just answered, just went over and all the kinks and quirks, then you got a whole new batch of students where you had to start from square one” (ID8)]. In this unit, they used less intensive behaviors to diminish the implementation burden.

Interviewees’ comments suggested that super users’ role engagement was primarily shaped by two contextual factors related to managers’ actions at the frontlines of the EHR implementation. First, super users’ engagement was shaped by the process through which they were selected. On Unit 1, where nurse managers asked super users to volunteer for the role, individuals experienced greater role engagement because they chose to participate. Through the process of deciding to become a super user, super users manifested a willingness to expand their role identity to include that of super user. Actively identifying as a super user, in turn, led to an increased sense of engagement, illustrated by one super user who said:

“[The Nurse Manager] just had us volunteer for it…she didn’t pick us, we volunteered for it… and I feel like me and [two super users’ names] were open to anything and I was trying to be the most positive, like [the EHR’s] great” (ID10).

On Unit 2, where nurse managers appointed super users because of their technological savvy, individuals were less engaged with the role after having the identity of super user thrust upon them. For example, when a super user on Unit 2 was asked how he was selected, he responded that the managers selected the super users “without much explanation.” Being excluded from the decision-making process led super users to passively assume the role rather than actively identify with it. As a visible sign of resistance to this new identity, one super user exclaimed to another clinician: “I’m not wearing that thing!” in reference to the purple-colored shirt that identified individuals as super users.

The second factor that modified super users’ role engagement was the implementation climate, or employees’ shared perceptions of the importance of innovation implementation in the organization [44], that was fostered by nurse managers. On Unit 1, nurse managers created a stronger implementation climate by actively supporting the hospital’s implementation policies, rewarding extra-role behaviors (i.e., behaviors that went above and beyond the role description), and modeling proactive behaviors. For example, the nurse manager on Unit 1 recounted her modeling efforts:

“If there was somebody who I thought might be struggling and not want to ask… I’d go up to them and I would just ask them to show me things. And when they couldn’t, I would just kind of say to them, ‘you know this [technique] is helpful’” (ID1).

In contrast, on Unit 2, nurse managers created a weaker implementation climate by passively complying with the hospital’s implementation practices, failing to reward extra-role behaviors, and modeling reactive behaviors. As an example of the managers’ reactive approach, on the first night of the EHR implementation, Unit 2’s managers had not anticipated the level of technical support the staff would need, leaving the unit understaffed. Although the assistant manager eventually moved to the night shift to provide additional support, the initial damage to morale had already been done, as expressed by the manager:

“They were very negative, nights. But for I think good reason – our miss [not planning for appropriate staffing levels]… started [the EHR implementation] with a bad taste. They thought, ‘well they’re not gonna be here to support us’… they just carried over this orphan mentality. ‘We’re not taken care of here’” (ID2).

Moreover, the nurse managers on Unit 2 contributed to a weak implementation climate by not always holding staff accountable for the changes that needed to be made [e.g., “I’m trying to give them a break because we don’t want to whip them” (ID11)].

### Effect of unit–super user differences on implementation

We found a significant difference in IS proficiency between units. Greater IS proficiency at follow-up was apparent for clinicians on Unit 1, where super users used more effort-intensive behaviors to foster implementation success, compared with Unit 2 (mean IS proficiency score = 3.25 versus 2.58, respectively, P-value = 0.003). This difference persisted even after adjusting for clinicians’ IS proficiency at baseline (P-value from adjusted analysis = 0.03).

## Discussion

The literature on EHR implementation highlights the importance of super users and indicates that their effectiveness in supporting the implementation process can vary. We found that four key behaviors were employed by super users on the unit with greater implementation success. These were: proactivity, depth of explanation, framing, and information-sharing. Furthermore, we found that these behaviors were modified by different levels of role engagement by the super users and varied as a function of how managers selected super users and shaped the implementation climates in which super users operated.

Our findings advance implementation science, the scientific study of influences on healthcare professional and organizational behavior with respect to promoting the systematic uptake of research findings into routine practice [45], by identifying the behaviors that super users use to influence other healthcare professionals during the implementation of EHRs. By identifying the behavioral mechanisms of super users’ influence, we add to a growing body of literature that highlights the significant effect of social influence on individuals’ acceptance of technology [46], but that had hitherto failed to elucidate the specific processes by which individuals influence other employees’ beliefs and behaviors [22]. This study also contributes to a more nuanced understanding of super users, whose role had previously only been discussed in a positive light, by highlighting that super users behave in ways that can positively *and* negatively affect implementation. In light of this finding, training programs for super users should not only focus on instilling positive behaviors, but train against negative behaviors.

This work also highlights the importance of engaging super users. Motivating employees to engage in their work is a classic problem in organizations [47], particularly for demanding roles like that of a super user, in which individuals must simultaneously balance multiple roles (e.g., end user of the EHR, trainer of the EHR), multiple constituencies (e.g., peers, supervisors), and multiple tasks (e.g., patient care, EHR technical support). Although leveraging individuals who occupy such “boundary spanning” roles may be critical to increasing user engagement with health IT [48], our findings highlight the need to engage the super users themselves, given the potential for their behaviors to both help, and hinder, implementation efforts.

Frontline managers can help foster super users’ engagement using two strategies. First, in terms of the selection process, managers should allow super users to volunteer for the position since we find that the choice to participate is a greater predictor of super users’ effectiveness than their technological savvy. This finding is supported by prior research by Halbesleben et al. [17], which found that super users’ previous experience with the information system (i.e., technological savvy) was not a significant predictor of employee attitudes about the technology. Choice, on the other hand, is a well-documented antecedent of engagement [49] and thus should be promoted by involving potential super users in the decision-making process about assuming this role. Second, managers should create an implementation climate supportive of change in order to augment super users’ positive impact. Past research has suggested that managerial support for implementation of an innovation is critical for implementation success [44], and we see that in the case of EHR implementation as well.

Our findings should be interpreted in light of several limitations. First, given the myriad factors that affect the EHR implementation process, we acknowledge that our focus on the role of super users is only one piece of a very complex puzzle, and that enabling the super user role may be a necessary – but certainly not sufficient – condition for fostering implementation success. Future research should assess the relative impact of super users and how the various factors that affect EHR implementation interact. Second, although our comparative case study allowed us to conduct an in-depth investigation of *how* super users influence implementation success, our focus on two units in the same hospital limits the generalizability of the findings. Studying units within the same hospital had the advantage of standardizing many of the organizational factors that might affect implementation outcomes (e.g., organizational culture, senior management support, training) [8]; the identified behaviors may differ in effectiveness in other organizational contexts. Third, our examination of the relationship between clinicians’ IS proficiency at follow-up and the clinical unit in which they worked was limited by a small sample size (which prevented us from adjusting for clinicians’ demographics due to limited power) and the use of observational data (which undermined our ability to make causal claims about how super users foster implementation success). Moreover, since our examination of super users’ behaviors was qualitative and not quantitative in nature, we were unable to directly test the relationship between super users’ behaviors and implementation outcomes. Future work could build on our study by using randomized trials to test how super users’ selection and behaviors affect implementation outcomes. Lastly, although the study was longitudinal, our nine month data collection period was relatively brief. We chose to focus on the early stage of implementation because it is when disruptions in processes and loss in productivity compel users to look most to others for guidance [50]. Our implementation outcome of interest, IS proficiency, represents a first stage measure that is expected to be salient in the early stages of a new behavior [46]. As EHRs are continuously updated and optimized, however, it will be important for future work to explore how super users help foster sustained use of technology over time.

## Conclusions

This study deepens our understanding of the role of super users in EHR implementation by identifying the specific behaviors that super users used to influence their peers. The unit in which super users were more engaged (i.e., were more proactive, provided more comprehensive explanations for their actions, used positive framing, and shared information more freely), experienced greater implementation success, as indicated by clinicians’ IS proficiency. To promote super users’ engagement with the role, our results suggest that frontline managers should allow super users to volunteer for the role and create an implementation climate supportive of change.
